# The isolation strategy and chemical analysis of oil cells from Asari Radix et Rhizoma

**DOI:** 10.1186/s13007-024-01184-5

**Published:** 2024-05-17

**Authors:** Haibo Hu, Guangxue Liu, Yaoli Li

**Affiliations:** 1 National Engineering Research Center for Modernization of Traditional Chinese Medicine-Hakka Medical Resources Branch, School of Pharmacy, Gannan Medical University, Ganzhou, 341000 China; 2 School of Pharmaceutical Sciences, Peking University, Beijing, 100191 China

**Keywords:** Oil cell, Plant single cell, Single-cell isolation, Single-cell analysis, Asari Radix et Rhizoma, Xixin, Beixixin, *Asarum heterotropoides* var. *mandshuricum*, Cell picking, Laser capture microdissection

## Abstract

**Background:**

Single-cell analysis, a rapidly evolving field, encounters significant challenges in detecting individual cells within complex plant tissues, particularly oil cells (OCs). The intricate process of single-cell isolation, coupled with the inherent chemical volatility of oil cells, necessitates a comprehensive methodology.

**Results:**

This study presents a method for obtaining intact OC from Asari Radix et Rhizoma (ARR), a traditional herbal medicine. The developed approach facilitates both qualitative and quantitative analysis of diverse OCs. To determine the most reliable approach, four practical methods—laser capture microdissection, micromanipulation capturing, micromanipulation piping, and cell picking—were systematically compared and evaluated, unequivocally establishing cell picking as the most effective method for OC isolation and chemical analysis. Microscopic observations showed that OCs predominantly distribute in the cortex of adventitious and fibrous roots, as well as the pith and cortex of the rhizome, with distinct morphologies—oblong in roots and circular in rhizomes. Sixty-three volatile constituents were identified in OCs, with eighteen compounds exhibiting significant differences. Safrole, methyleugenol, and asaricin emerged as the most abundant constituents in OCs. Notably, cis-4-thujanol and tetramethylpyrazine were exclusive to rhizome OCs, while isoeugenol methyl ether was specific to fibrous root OCs based on the detections. ARR roots and rhizomes displayed marked disparities in OC distribution, morphology, and constituents.

**Conclusion:**

The study highlights the efficacy of cell picking coupled with HS–SPME–GC–MS as a flexible, reliable, and sensitive method for OC isolation and chemical analysis, providing a robust methodology for future endeavors in single-cell analyses.

**Supplementary Information:**

The online version contains supplementary material available at 10.1186/s13007-024-01184-5.

## Background

The extensive molecular profiling analysis of single cells has garnered significant interest, and the analysis of chemical substances at the single-cell level is gradually assuming a pivotal role in life science research [1–4]. The ongoing exploration of metabolic heterogeneity between different cells continues to advance our understanding of physiological and biological phenomena and their applications [5–7]. Presently, the foremost challenge in single-cell component analysis stems from factors like small cell size and a large number of molecules at varying concentrations [8]. However, advancements in science and technology have led to the development of analytical techniques with detection sensitivity at the single-cell level, making single-cell analysis feasible. Techniques such as fluorescence [9], capillary electrophoresis (CE) [10], chromatography-mass spectrometry (MS) [11, 12], CE-MS [13], microelectrodes [14], microfluidics [15], NMR spectroscopy [16] and Raman spectroscopy [17], among others, have enabled researchers to explore the intricate world of single-cell analysis. Mass spectrometry has rapidly evolved into a powerful method in chemical analysis, owing to its high sensitivity, excellent specificity, label-free nature, and information-rich features [4]. Notably, electrospray ionization (ESI)/nano ESI MS [13], MALDI-MS [18] and secondary ion mass spectrometry [19] are conventional MS-based techniques for chemical analysis. Recent developments in ambient MS [20] offer a promising avenue for directly detecting compounds within living cells. However, its limited injection volume and lower sensitivity have constrained its widespread application, preventing it from competing with conventional mass spectrometry in terms of popularity [4, 20, 21]. Unless utilizing direct detection methods such as ambient MS [20], single-cell analysis techniques typically require the separation or isolation of cells. As of now, the precise composition of a single oil cell and the variations in composition among different OCs within a whole plant remain elusive.

To address these inquiries, the traditional Chinese herb, Asari Radix et Rhizoma was selected as our study subject. According to the Chinese pharmacopeia, ARR is derived from the dry roots and rhizomes of three *Asarum* plants. Among these, the most commonly used variety is *Asarum heterotropoides* Fr. Schmidt var. *mandshuricum* (Maxim.) Kitag., known as Xixin or Beixixin in Chinese, which was chosen for this study. This herb holds historical significance, being documented in *Shennong Bencaojing* during 25–220 AD, and it is widely used in China to treat various ailments such as cold, cough, sinusitis, toothache, and rheumatic arthralgia, also possessing antiseptic and odoriferous properties [22–24]. ARR has found applications in the food industry as an additive [25] and shows potential for development as a pesticide and larvicide due to its anti-phytopathogenic and larvicidal activities [26, 27]. Our objective was to isolate intact OCs from ARR, investigate their distribution and morphological characteristics, and analyze their chemical components. In summary, four approaches were evaluated to obtain single ARR OCs for chemical analysis, and the effective sample preparation technique, solid-phase microextraction (SPME) [28–30] was carried out to enrich the components from single OCs. We herein performed a headspace-SPME-gas chromatography-mass spectrometry (HS–SPME–GC–MS) combined technology for OC chemical analysis, in which all MS data were processed by XCMS [27, 31–35] to reduce manual comparison errors. Then, their structures were elucidated according to ions and retention index (RI) compared with standards and database. Afterward, multivariate statistical analyses [36–38] were performed to obtain reliable qualitative and quantitative discrimination of OCs compounds in different ARR parts. The aim is to establish a reliable method for the single-cell separation, purification, and composition analysis of OCs, and to provide references for single-cell studies of plants.

## Results

### Oil cell distribution

**Fig. 1:**
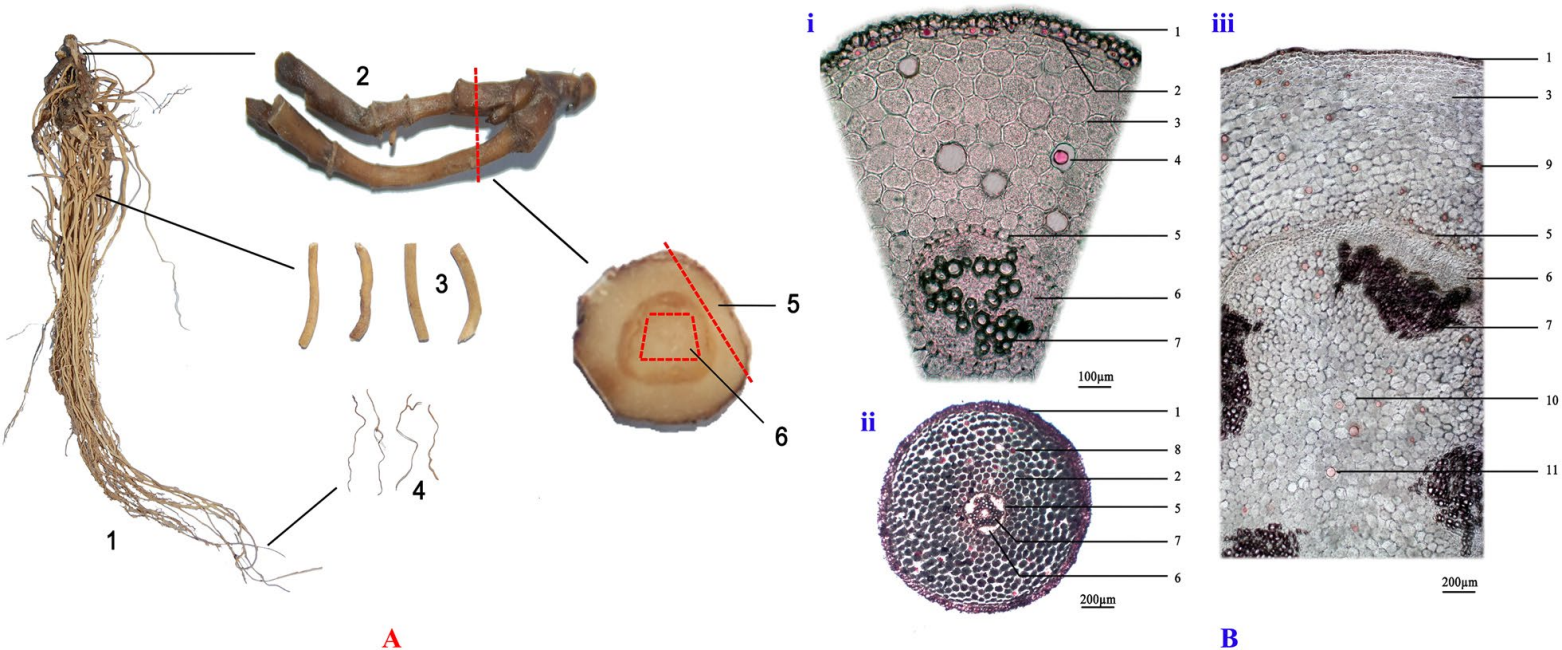
Tissue separation (**A**) and cross-section observation of Asari Radix et Rhizoma (**B**). A1: Asari Radix et Rhizoma, A2: rhizome, A3: adventitious roots, A4: fibrous roots, A5: cortex part, A6: pith. B-i: adventitious root interrupted cross-section, B-ii: fibrous root cross-section, B-iii: rhizome cross-section. B1: epidermis, B2: outer cortex, B3: cortex, B4: cortical oil cells in the adventitious root, B5: endodermis, B6: phloem, B7: xylem, B8: cortical oil cells in fibrous roots, B9: rhizome cortical oil cells, B10 pith, B11 rhizome pith oil cells

The materials of ARR encompass rhizomes, adventitious roots, and fibrous roots. Cross-sectional observations were conducted to examine the distribution of OCs, as depicted in Fig. 1. The microstructure of adventitious roots and fibrous roots primarily comprised the epidermis, cortex, and vascular column. In adventitious roots, the outermost layer was covered by the residual thickened epidermal cells, referred to as metaderm. These cells formed a layer of approximately circular cells arrayed tangentially, featuring slightly thickened cell walls and smaller dimensions than normal epidermal cells. Approximately 10–17 layers of cortical cells were present, with the outer 2–3 layers tangentially extending, some of which differentiated into OCs arranged in a circular pattern. The inner cortex cells, exhibiting distinct intercellular spaces, were round in shape with larger diameters, housing numerous scattered OCs. Endothelial cells displayed visible Casparian dots outside of pericycle cells (1–2 layers). In the cylinder, the primary xylem developed in a 2–4 prototype, and the 1–3 parenchyma cells (significantly larger than the surrounding phloem cells) were positioned at the center of the phloem bundle, while their long diameter was notably smaller than the maximum catheter diameter.

The rhizome was primarily composed of four parts: epidermis, cortex, cylinder, and pith. The epidermal cells were arranged in a single layer, with very few cells specialized into OCs. There were 15–22 rows of cortical cells, including scattered round OCs, and the outer cortex consisted of 1–2 layers with few OCs. Importantly, a significant number of OCs were distributed in the broad cortex and well-developed central pith, and few stone cells were observed around the phloem and xylem. In summary, the sectional study revealed that ARR OCs were mainly distributed in the cortex of the fibrous root and adventitious roots, as well as the pith and cortex of rhizomes.

### Cell isolation approaches

**Fig. 2:**
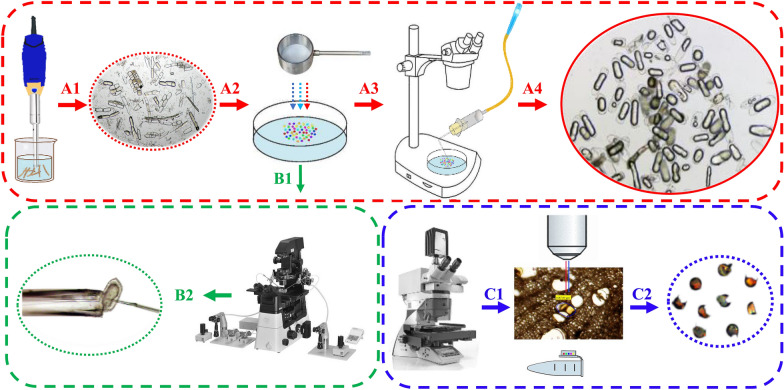
Physical isolation methods for separating and purifying single oil cells. **A** Cell picking (smashing tissues, sieving, manual selection of single oil cell); **B** Micromanipulation capturing and piping (with glass needle for oil cells and their inclusion); **C** Laser microdissection (broken oil cells and their contents)

In the analysis of compounds within single cells, concerns often arise regarding the potential impacts of the isolation and sampling procedures. Some studies have employed enzymes like cellulase, hemicellulose, protopectinase, polygalacturonase to digest tissues and filter to obtain OC or inclusion, as well as oil bodies from Japanese soybeans and idioblast cells from the avocado fruit [39, 40]. However, these enzymes may affect cellulose and polysaccharides in cell walls, causing inclusion overflow and potentially altering the chemical compositions inside cells. Without the single-cell selection process, the cells may not be intact and pure for compound analysis. Therefore, four single-cell methods were utilized in this study to obtain intact OCs or their inclusion, including laser capture microdissection (LCM), micromanipulation capturing, micromanipulation piping, and cell picking (Fig. 2). The results indicated most of these methods were practical to obtain OCs, each with its own set of advantages and disadvantages.

LCM, although effective in cutting tissues with a laser to separate OCs, often resulted in broken cells due to the required sectioning progress and laser damage. To validate this hypothesis, different tissues (the epidermis, cortex, phloem, and xylem) and OCs from adventitious roots were lasered and analyzed by HS–SPME–GC–MS. Metabonomic comparisons showed these tissues and OCs had similar chemical compositions according to the total ion chromatograms, indicating that LCM might not be suitable for single OC analysis (Additional file 1). Microscopic cell operations were also evaluated to capture OCs in the suspension after physically homogenized tissues. However, transferring the cells to another container for MS detection proved challenging due to the limited scope and area of the platform. Furthermore, aspirating the contents from OCs with a micromanipulation needle was attempted, but the thickened cell wall and semisolid inclusion posed difficulties in pipetting the content. The suction range was also limited, making it challenging to extract the contents with controlled power. Previous reports [41] showed that using a microsyringe to pipette the content of OCs in fresh leaf slices of *Tasmannia lanceolata* yielded only two detected and identified components, suggesting that piping OC inclusion might not provide sufficient information for compound analysis.

**Fig. 3:**
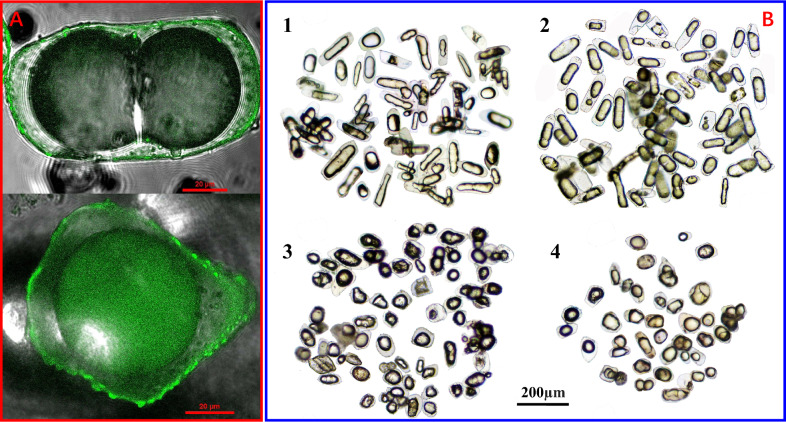
Laser confocal observation (**A**) and light micrograph (**B**) of oil cells in adventitious roots (1), fibrous roots (2), cortex part (3), and pith part (4) of rhizomes from Asari Radix et Rhizoma

In contrast, cell picking successfully provided significant amounts of different OCs from various parts of ARR, making it highly recommended for obtaining a single plant cell. The process involved four main steps (Fig. 2A): first, a blade was used to cut different tissues with OCs under a stereomicroscope, resulting in four tissues including the cortex of fibrous roots (XW), the cortex of adventitious roots (XG), the pith (SUI) and cortex (PI) of the rhizomes; second, tissues were physically homogenized to be suspended as a mixture with cells; third, physical micron screens with bore diameters of 300 µm and 80 µm were used to eliminate tissues and cell residues, with the OCs retained on the 80 µm cell screen and transferred into a suspension; finally, a self-made cell picking tool with a top glass needle and a contamination protector was used to transfer single OCs for further analysis. The results (Fig. 3) provided a completely feasible way for single OC separation, proving useful for other types of plant cells. Although, the OCs were isolated and analyzed in water, potentially affecting the polar or water-soluble compounds, the volatile components should remain inside the intact OCs. Therefore, cell picking was deemed the most effective method for the volatile chemical analysis of oil cells in this study.

### Diversity of oil cells

**Fig. 4:**
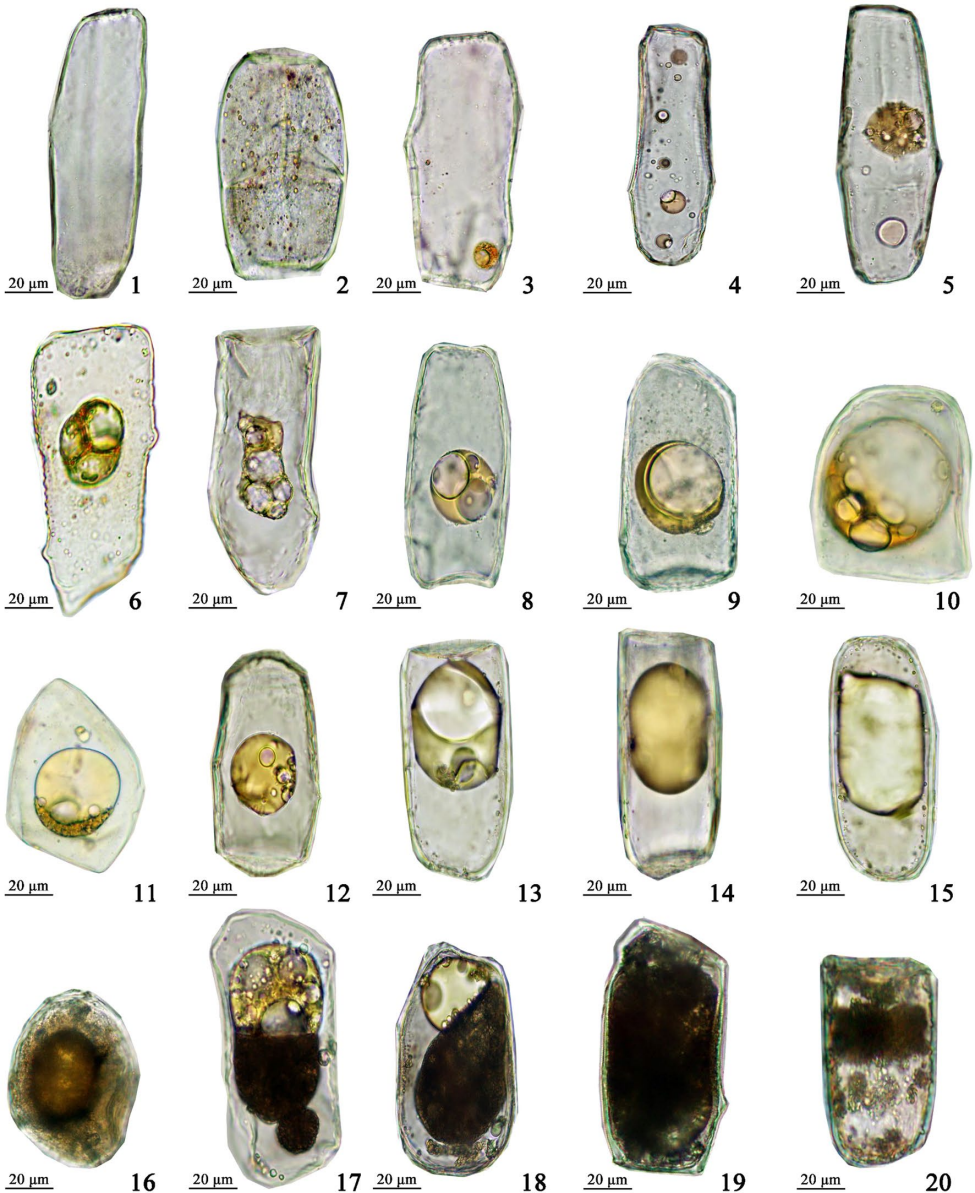
Morphology of oil cells at different developmental stages from Asari Radix et Rhizoma. 1: Oil-free period, 2–4: oil-droplet period, 5–11: oil-accumulation period, 12–15: oil- saturation period, 16–20: oil-degradation period

To observe the variety of OCs, micro-examination was carried out while physically homogenizing ARR tissues and staining them with Sudan III in these suspensions. In Fig. 4, the oil or inclusions were stored in oil bodies or cysts of OCs, displaying various shapes. Similar to the oil cells in other plants [42, 43], five different stages for oil development and accumulation can be observed in ARR, according to the morphological characteristics of oil bodies and cysts, including the oil-free, oil-droplet, oil-accumulation, oil-saturation, and oil-degradation periods. Given the feasibility of operation, this study mainly focused on the sampling and analysis of OCs in oil saturation, where large central droplets took shape and persisted for the longest duration, consistent with their most common presence.

The integrity of the picked oil cells was verified using optical microscopes, revealing two morphologies—oblong and circular (Fig. 3). Oblong OCs were observed in roots, while round OCs were found in rhizomes. The long diameter measurements of 100 OCs were randomly conducted, revealing significant differences with diameters of 135.88 μm, 140.67 μm, 79.80 μm, and 77.12 μm for OCs in XG, XW, PI, and SUI, respectively. These isolated OCs were examined for intact morphology under the light microscope (Fig. 3B). Laser confocal observations were also performed for all types of OCs, leveraging their autofluorescence that highlights substances inside plant cells, including suberin, lignin, etc. [44] The results indicated that auto-fluorescent substances of OCs were primarily distributed on the outer periphery (Fig. 3A). These luminous compounds demonstrated the integrity of the three-dimensional morphology of cell walls and internal capsules. Thus, the process of cell picking for isolating oil cells successfully yielded intact OCs, and observations across multiple samples confirmed the reliability of this method.

### HS–SPME–GC–MS methodology

In this study, HS–SPME–GC–MS was employed to concentrate and detect the volatile chemicals from OCs. The GC–MS condition was optimized based on our previous research [45]. For HS-SPME, both the temperature and time significantly influenced the evaporation, sampling, and detection of chemicals. Consequently, HS-SPME conditions were evaluated using the peak area of five main constituents (3,5-dimethoxytoluene, safrole, methyleugenol, 2,3,5-trimethoxytoluene, and asaricin), with the detailed parameters provided in “HS–SPME–GC–MS condition”. Microscopic examination was performed on the utilized OC samples to check the effectiveness of HS-SPME extraction, revealing deflated OCs with evaporated contents. Furthermore, the detection method was validated for linearity, accuracy (recovery), selectivity, repeatability, intermediate precision, LOD (Limit of Determination), and LOQ (Limit of Quantitation), following AOAC guidelines [46]. Calibration curves, derived from all ten standards, exhibited linearity with an R^2^ ≥ 0.98. Accuracy (recovery) was confirmed by adding the standards at high, middle, and low concentration levels (n = 3, equivalent to 80%, 100%, and 120% of the content of each reference substance in the materials) into a selected sample, achieving a recovery higher than 95%. Selectivity, assessed through the resolution of standard peaks in the GC chromatogram, exceeded ≥ 2. Repeatability and intermediate precision, based on six parallel measurements of XG samples, showed satisfactory repeatability with all RSDs of five selected constituents less than 3%, including RSDs of peak area as 1.91%, 1.68%, 1.80%, 1.93%, and 2.60%, respectively. For LOD and LOQ, 1, 10, 50, and 100 cells were evaluated through the MS signals, wherein even a single cell exhibited sufficient intensity and sensitivity, reaching × 10^7^ CPS (counts per second). Acknowledging the challenges associated with the low number of single cells for each detection, ten-cell sampling was conducted for each test to ensure the reliability and representativeness of the compounds in OCs.

### Qualitative analysis

**Fig. 5:**
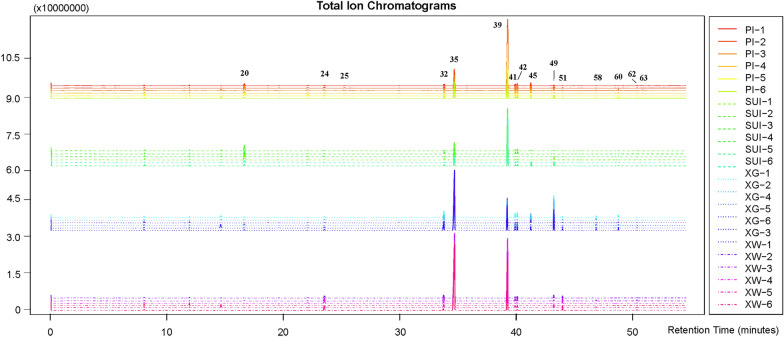
Total ion chromatogram (TIC) of oil cells from rhizome cortex (PI1-6), rhizome pith (SUI1-6), adventitious root cortex (XG1-6), and fibrous root cortex (XW1-6) of Asari Radix et Rhizoma. The names of marked compounds are listed in Table 1

For the analysis, each type of OCs was sampled six times and analyzed to identify their components, including OCs in XG, XW, SUI, and PI. Figure 5 illustrates the total ion chromatography of all samples (10 OCs per sample), generated by XCMS data alignment. All MS data were subjected to the peak area normalization, and the average identified peak areas of each type of OC accounted for 92.71%, 91.09%, 88.07%, and 89.82% of their total peak areas in XG, XW, SUI, and PI, respectively, indicating effective separation and characterization of the majority of components in ARR OCs. The spectra, labeled with different colors, exhibited significant differences. A total of 63 volatile components were identified, primarily belonging to monoterpenes and phenylpropanoids. Among them, 60 compounds were detected in XG, 61 in XW, 62 in SUI, and 61 in PI, with 60 common components detected in all OCs.

Relative contents were compared using the area normalization method, revealing significant differences among the OCs (Table 1). In XG-OCs, the primary compounds were safrole (47.97%), methyleugenol (13.23%), asaricin (10.89%), 3,5-dimethoxytoluene (4.67%), and croweacin (2.27%), while XW-OCs predominantly contained safrole (46.4%), methyleugenol (26.6%), acetophenone (5.64%), estragole (2.08%), and 3,5-dimethoxytoluene (2.07%). Remarkably, the contents in rhizome OCs differed significantly from those in roots. SUI-OCs exhibited methyleugenol (48.1%), safrole (11.1%), eucarvone (8.12%), and 3,5-dimethoxytoluene (2.21%), and PI-OCs showed methyleugenol (49.41%), safrole (15.18%), eucarvone (3.79%), 2,3,5-trimethoxytoluene (2.74%), croeacin (2.57%), 3,5-dimethoxytoluene (2.53%), and 3,4,5-trimethoxytoluene (2.08%). According to a previous report, the volatile compounds in ARR herb included methyleugenol/3,4,5-trimethoxytoluene (mixed), safrole, 3,5-dimethoxytoluene, eucarvone, 2,3,5-trimethoxytoluene, croweacin/asaricin (mixed), and 43 other compounds ranked from highest to lowest abundance [47]. The main compounds identified in the tiny individual oil cells closely corresponded to those found in ARR, demonstrating the validity of this approach. This finding directly addresses the inquiries posed in the introduction section regarding the feasibility and efficacy of the method employed in this study. Moreover, 3,4,5-trimethoxytoluene and methyleugenol were separated well here in GC–MS, as well as croweacin and asaricin, due to the optimized conditions in our experiment.

**Table 1:** Volatile compounds and their relative contents in different oil cells of Asari Radix et Rhizoma

No.	Compound	Formula	Molecule weight	RT (min)	Identification		Peak area (%)			
					RI	Spectra	PI	SUI	XG	XW
**1**	3-Carene	C_10_H_16_	136.2340	5.995	**1157**	MS^a^	0.42	0.76	0.11	0.24
**2**	α-Phellandrene	C_10_H_16_	136.2340	6.310	**1173**	MS^a^	0.38	0.51	0.17	0.17
**3**	D-Limonene	C_10_H_16_	136.2340	7.085	**1209**	MS^a^	0.10	0.1	0.05	0.09
**4**	β-Phellandrene	C_10_H_16_	136.2340	7.235	**1215**	MS^a^	0.20	0.23	0.11	0.01
**5**	p-Cymene	C_10_H_14_	134.2182	8.725	**1278**	MS^a^	0.13	0.14	0.05	0.03
**6**	Terpinolene	C_10_H_16_	136.2340	9.050	**1291**	MS^a^	0.22	0.36	0.03	0.03
**7**	Tridecane	C_13_H_28_	184.3614	9.470	**1308**	MS^a^	0.03	0.04	0.03	0.03
**8**	6-Methyl-5-hepten-2-one	C_8_H_14_O	126.1962	10.39	**1345**	MS^a^	0.02	0.02	0.02	0.01
**9**	Nonanal	C_9_H_18_O	142.2386	11.780	**1401**	MS^a^	0.15	0.28	0.18	0.16
**10**	Tetradecane	C_14_H_30_	198.3880	11.960	**1407**	MS^a^	0.23	0.26	0.16	0.14
**11**	Limonene monoxide	C_10_H_16_O	152.2334	12.940	**1439**	MS^a^	0.05	0.05	0.25	0.02
**12**	2-Methyldodecane	C_15_H_32_	212.4146	13.505	**1458**	MS^a^	0.02	0.05	0.02	0.02
**13**	*cis*-4-Thujanol	C_10_H_18_O	154.2493	13.749	**1466**	MS^a^	0.05	0.05	NT	NT
**14**	Tetramethylpyrazine	C_8_H_12_N_2_	136.1943	13.904	**1471**	MS^a^	NT	0.06	NT	NT
**15**	Ethylhexanol	C_8_H_18_O	130.2279	14.655	**1496**	MS^a^	0.04	0.04	0.03	0.08
**16**	Decanal	C_10_H_20_O	156.2652	14.955	**1504**	MS^a^	0.07	0.2	0.10	0.12
**17**	Pentadecane	C_15_H_32_	212.4146	15.085	**1506**	MS^a^	0.94	1.26	1.27	0.58
**18**	Camphor	C_10_H_16_O	152.2334	15.305	**1511**	MS^a^	0.13	0.07	0.03	0.01
**19**	1-Pentadecene	C_15_H_30_	210.3987	16.225	**1530**	MS^a^	0.08	0.05	0.09	0.01
**20**	**Eucarvone**	C_10_H_14_O	150.2176	17.075	**1548**	MS^b^	3.79	8.12	1.19	0.3
**21**	Terpinen-4-ol	C_10_H_18_O	154.2493	19.685	**1602**	MS^a^	0.11	0.1	0.04	0.04
**22**	Pivalone	C_9_H_18_O	142.2386	19.930	**1605**	MS^a^	0.12	0.15	0.07	0.04
**23**	Acetophenone	C_8_H_8_O	120.1485	22.510	**1646**	MS^a^	1.52	1.77	0.79	5.64
**24**	Estragole	C_10_H_12_O	148.2017	23.945	**1668**	MS^a^	1.18	1.18	1.16	2.08
**25**	**L-Borneol**	C_10_H_18_O	154.2493	25.645	**1695**	MS^b^	0.75	0.53	0.28	0.17
**26**	2-Methylundecanal	C_12_H_24_O	184.3184	27.670	**1726**	MS^a^	0.06	0.11	0.11	0.06
**27**	Valencane	C_15_H_28_	208.3828	27.800	**1728**	MS^a^	0.16	0.24	0.16	0.13
**28**	3-Methylheptadecane	C_18_H_38_	254.4943	31.070	**1778**	MS^a^	0.01	0.01	0.02	0.01
**29**	Phytan	C_20_H_42_	282.5475	31.515	**1785**	MS^a^	0.03	0.06	0.05	0.03
**30**	Sabinyl acetate	C_12_H_18_O_2_	194.2701	32.465	**1800**	MS^a^	0.06	0.06	0.01	0.02
**31**	p-Mentha-1(7),8-dien-2-ol	C_10_H_16_O	152.2334	33.595	**1829**	MS^a^	0.06	0.04	0.02	0.01
**32**	**3,5-Dimethoxytoluene**	C_9_H_12_O_2_	152.1904	34.225	**1845**	MS^b^	2.53	2.21	4.67	2.07
**33**	p-Cymen-8-ol	C_10_H_14_O	150.2176	34.525	**1852**	MS^a^	0.05	0.08	0.01	0.01
**34**	Nerylacetone	C_13_H_22_O	194.3132	34.645	**1856**	MS^a^	0.09	0.29	0.15	0.07
**35**	**Safrole**	C_10_H_10_O_2_	162.1852	35.125	**1868**	MS^b^	15.18	11.1	47.97	46.4
**36**	Trans-isosafrole	C_10_H_10_O_2_	162.1852	37.515	**1939**	MS^a^	0.01	0.02	0.03	0.03
**37**	Ethane-1,2-diyl bis(2-methylbutanoate)	C_12_H_22_O_4_	142.2386	38.405	**1970**	MS^a^	0.05	0.06	0.04	0.02
**38**	2-Allyl-1,4-dimethoxybenzene	C_11_H_14_O_2_	178.2277	39.000	**1990**	MS^a^	0.07	0.15	0.07	0.02
**39**	**Methyleugenol**	C_11_H_14_O_2_	178.2277	39.645	**2013**	MS^b^	49.41	48.1	13.23	26.6
**40**	Isosafrole	C_10_H_10_O_2_	162.1852	39.860	**2020**	MS^a^	0.05	0.05	0.03	0.16
**41**	**3,4,5-Trimethoxytoluene**	C_10_H_14_O_3_	182.2164	40.340	**2037**	MS^b^	2.08	1.55	1.46	0.68
**42**	**2,3,5-Trimethoxytoluene**	C_10_H_14_O_3_	182.2164	40.525	**2043**	MS^b^	2.74	1.69	1.59	0.91
**43**	3,4,5-trimethoxy- Benzoic acid	C_10_H_12_O_5_	212.1993	40.705	**2049**	MS^a^	0.04	0.02	0.02	0.01
**44**	Isoeugenol methyl ether	C_11_H_14_O_2_	178.2277	41.630	**2081**	MS^a^	NT	NT	NT	0.04
**45**	Croweacin	C_11_H_12_O_3_	192.2112	41.680	**2083**	MS^a^	2.57	1.36	2.27	0.03
**46**	3-Allyl-6-methoxyphenol	C_10_H_12_O_2_	164.2011	43.115	**2132**	MS^a^	0.02	0.02	0.06	0.02
**47**	n-Pentadecanol	C_15_H_32_O	228.4140	43.335	**2140**	MS^a^	0.15	0.22	0.02	0.1
**48**	Methyl isoeugenol	C_11_H_14_O_2_	178.2277	43.480	**2145**	MS^a^	0.21	0.23	0.06	0.11
**49**	Asaricin	C_11_H_12_O_3_	192.2112	43.670	**2152**	MS^a^	1.33	1.79	10.89	1.34
**50**	4'-Methoxypropiophenone	C_10_H_12_O_2_	164.2011	43.815	**2157**	MS^a^	0.06	0.21	0.09	0.02
**51**	**Elemicin**	C_12_H_16_O_3_	208.2536	44.415	**2177**	MS^a^	0.37	0.29	0.76	0.98
**52**	Veratric acid	C_9_H_10_O_4_	182.1733	44.660	**2186**	MS^a^	0.04	0.04	0.04	0.02
**53**	γ-asarone	C_12_H_16_O_3_	208.2536	45.085	**2201**	MS^a^	0.04	0.07	0.07	0.03
**54**	4,8,12-Tetradecatrienal, 5,9,13-trimethyl-	C_17_H_28_O	248.4036	45.445	**2213**	MS^a^	0.03	0.15	0.14	0.1
**55**	Cyclohexanone, 2 -(hydroxymethylene) -3-methyl-6-(1-methylethyl)-	C_11_H_18_O_2_	182.2594	45.825	**2226**	MS^a^	0.20	0.21	0.11	0.1
**56**	2,4-Di-tert-butylphenol	C_14_H_22_O	206.3239	46.070	**2235**	MS^a^	0.16	0.19	0.13	0.1
**57**	Myristicin	C_11_H_12_O_3_	192.2112	46.965	**2265**	MS^a^	0.04	0.06	0.09	0.04
**58**	**3,4-Methylenedioxypropiophenone**	C_10_H_10_O_3_	178.1846	47.300	**2277**	MS^b^	0.26	0.12	0.82	0.24
**59**	2',4'-Dimethoxypropiophenone	C_11_H_14_O_3_	194.2271	47.815	**2295**	MS^a^	0.04	0.03	0.05	0.01
**60**	**Kakuol**	C_10_H_10_O_4_	194.1840	49.210	**2343**	MS^b^	0.40	0.27	0.79	0.13
**61**	Xanthoxylin	C_10_H_12_O_4_	196.1999	49.660	**2359**	MS^a^	0.03	0.04	0.02	0.01
**62**	Dibutyl phthalate	C_16_H_22_O_4_	278.3435	50.810	**2398**	MS^a^	0.28	0.39	0.28	0.33
**63**	2',4'-Dimethoxy-3'-methylpropiophenone	C_12_H_16_O_3_	208.2536	51.300	**2415**	MS^a^	0.18	0.11	0.15	0.04

*NT* not found

Standards were marked in bold

^a^Identification by the comparison of mass spectra in NIST14 library

^b^Identification via standards

### Differential component identification using XCMS

Untargeted metabolomics, known for its ability to analyze various metabolites, has found widespread application in comparing differences among multiple samples [48]. In this study, the online metabolomics analysis tool, XCMS [27, 31–35] was employed to align mass spectrometry (MS) ions and retention times for qualitative analysis of differential components across all samples. The aligned data included mass-to-charge ratio, retention time, P-value, Q value, and intensity of ions in each sample. For the OC data, ions with zero-value intensity were sieved as the components not present in the sample(s). In this analytical dataset, 354 ions displayed zero-intensity in one or more of the sample types. Three specific compounds giving rise to these ions were identified, including cis-4-thujanol, tetramethylpyrazine, and isoeugenol methyl ether. Among them, cis-4-thujanol was exclusively detected in rhizome PI- and SUI-OCs, tetramethylpyrazine only in SUI-OCs, and isoeugenol methyl ether in XW-OCs. However, considering the low peak areas (≤ 0.05%) of these three compounds, the detection limit may also be responsible for their absence in some related OCs.

### Multivariate statistical analysis

**Fig. 6:**
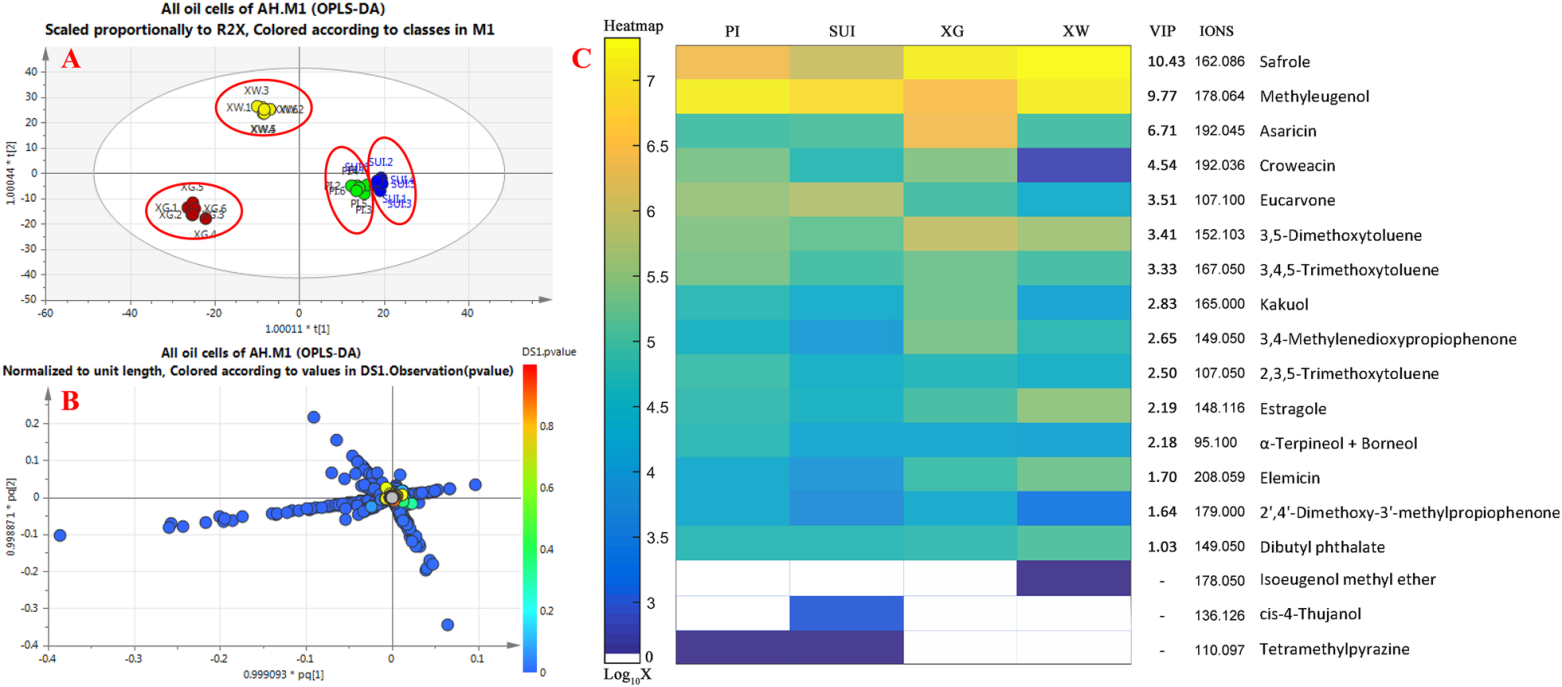
OPLS-DA scores plot (**A**), lording plot (**B**) of all MS data, and heatmap (**C**) of OCs’ markers (VIP > 1). The oil cells were from rhizome cortex (PI), rhizome pith (SUI), adventitious root cortex (XG), and fibrous root cortex (XW) of Asari Radix et Rhizoma

The data without zero values were considered as the ions from common components in all OCs. To compare their differences, various multivariate statistical analysis (MSA) methods [36, 37, 49] were introduced to establish a relationship model between component expression and samples. This facilitated prediction and judgment analysis for sample categorization, utilizing techniques such as principal component analysis (PCA), partial least-squares discrimination analysis (PLS-DA), and orthogonal partial least-squares discrimination analysis (OPLS-DA). The steps involved importing the data without zero values into SIMCA-P 14.0 software and fitting the solution via different models. The optimal model was constructed under OPLS-DA with the PAR model, achieving Q^2^ = 0.845, R^2^X = 0.896, and R^2^Y = 0.999. R^2^X and R^2^Y represented the interpretation rate of the X and Y matrices, respectively, while Q^2^ indicated the predictive ability of the model. In theory, the closer of R^2^ and Q^2^ values are to 1, the better the built model. As illustrated in Fig. 6a, the 24 samples are aggregated into four categories, indicating significant differences in their volatile components and the successfully established model.

Furthermore, the ion characteristics of rhizome pith and rhizome cortex OCs were relatively close, suggesting that their components were quite similar. VIP (variable importance for the projection) values were then calculated and compared for the influence strength and explanatory ability of composition expression on the classification and discrimination. Data with VIP value > 1.0 were considered significant differences. OPLS-DA generated a scatter-loading map by fitting the data without zero (Fig. 6b). In the dispersion map of ions, those farther from the sub-cluster typically had higher VIP values, indicating a greater chance of marker presence. In other words, ions scattered farther from the main cluster were more likely to be labeled. Fifteen biomarkers were identified with VIP > 1, including safrole, methyleugenol, asaricin, croeacin, eucarvone, 3,5-dimethoxytoluene, 3,4,5-trimethoxytoluene, kakuol, 2,3,5-trimethoxytoluene, 3,4-methylenedioxypropiophenone, estragole, l-borneol, elemicin, 2',4'-dimethoxy-3'-methylpropiophenone and dibutyl phthalate, with VIP values ranked from greatest to least (Fig. 6c).

### Semi-quantitative analysis

Semi-quantitative analysis was conducted by comparing ion intensities for the above 15 biomarkers in all OCs, as well as three differently distributed components. Relative contents were normalized and calculated as the average intensity of characteristic ions based on six parallel OC samples. In Fig. 6c, these characteristic ions, VIP values, and intensity differences were ranked for each compound, displayed in diverse colors along with their logarithm values. To enhance clarity, these 15 compounds and the three specific compounds were classified into three levels based on ion intensity, including high-level (ion intensity > 10^6^ cps), general level (10^5^–10^6^ cps), and low-level (< 10^5^ cps) contents. Three high-level components were safrole, methyleugenol, and asaricin. Safrole was predominantly distributed in root OCs (85.8%) while asaricin was prominent in adventitious root OCs (79.1%). The general-level components comprised of 3,5-dimethoxytoluene, eucarvone, 3,4,5-trimethoxytoluene, croweacin, estragole, dibutyl phthalate, 3,4-methylenedioxy-propiophenone, elemicin, kakuol, 2,3 5-trimethoxytoluene, and L-borneol. Most of these compounds had higher relative content in root OCs, including 3,5-dimeth-oxytoluene (69.7%), 3,4-methylenedioxypropiophenone (80.6%), estragole (74.7%), elemicin (83.9%), and kakuol (65.2). However, eucarvone (84.6%) and L-borneol (64.6%) were mainly found in rhizome OCs. The remaining compounds, 3,4,5-trimethoxytoluene, dibutyl phthalate, and 2,3,5-trimethoxytoluen showed even distribution. For low-level components, tetramethylpyrazine was only detected in rhizome OCs, cis-4-Thujanol only in pith OCs, and isoeeugenol methyl ether only in fibrous root OCs. Similar to the significant morphological differences, OCs of the root and rhizome exhibited substantial disparities in their components.

## Discussion

Current cell separation technologies fall into two main categories: physical properties-based separation and biological characteristics-based separation. The former methods include density gradient centrifugation, membrane filtration, and microchip-based capture platforms, while the latter involves affinity methods based on biological protein expression, fluorescence-activated cell sorting (FACS), and magnetic-activated cell sorting (MACS) [50–52]. Common techniques in this context include FACS [53], MACS [54], microfluidics [15], LCM [55–58], micromanipulation, and cell picking [52, 59]. High-throughput technology is often employed for separating plant protoplasts without cell walls [60], and LCM is utilized for obtaining cells or subcellular structures [58, 61–63]. However, limited progress has been made in isolating single intact cells from plants, particularly oil cells rich in volatile components.

Plant OCs, characterized by thickened cell walls [44, 64], pose challenges for separation and assembly using high-throughput methods like FACS and MACS, further exacerbated by the absence of corresponding probes. Density gradient centrifugation, membrane filtration, or microfluidics, with their stringent requirements for cell homogeneity, are also unsuitable for OC isolation. In contrast to these advanced technologies, cell picking enables the direct observation and imaging of individual plant cells under a stereomicroscope, facilitating precise isolation. While traditionally applied to manipulate small organisms, animal embryos, or egg cells, cell picking is seldom utilized for plant cells. Surprisingly, manual cell picking, particularly via mouth pipetting, accounted for 12% of single-cell isolation methods in a recent survey, ranking third after microfluidics (29%) and flow cytometry (41%) [65]. Despite its skill-dependent and labor-intensive nature, this conventional approach remains crucial in many laboratories, even those equipped with automated instruments [66]. In this study, various methods for oil cell isolation were evaluated, encompassing LCM, micromanipulation capturing, micromanipulation piping, and cell picking. The results indicate that both LCM and cell picking offer convenience for obtaining individual oil cells. However, for the chemical analysis of essential oil, cell picking proves to be significantly superior to the other methods.

For ARR, GC–MS and HS-GC–MS have been conducted on its various parts, including the whole plant, underground parts, and n-hexane extracts. These analyses led to the identification of approximately 80 components, with methyleugenol and safrole emerging as the most abundant chemicals. Both of them also serve as the primary bioactive components in ARR [39, 47, 67, 68]. The pharmacological activities of ARR roots and rhizomes have been reported to differ, primarily due to their distinct volatile chemical compositions [47, 69]. Despite this existing knowledge, there remains a scarcity of information regarding the differences in OC components between ARR roots and rhizomes. This highlights the importance of our study in uncovering and understanding the specific volatile chemical variations within the ARR OCs. However, the analysis method employed in this study, with ten-cell sampling for each detection, posed a limitation in assessing the chemical heterogeneity of different single cells derived from a given tissue. Nonetheless, the detection of a single OC here exhibited sufficient intensity and sensitivity, enabling this discernment, and this methodology supports further investigations into single-cell analysis.

## Conclusion

This study highlights the effectiveness of cell picking combined with HS–SPME–GC–MS as a flexible, reliable, and sensitive method for isolating intact oil cells and conducting a comparative chemical analysis. While acknowledging that cell picking has its drawbacks of being skill-dependent and labor-intensive, the technique demands a certain level of expertise or practice. Additionally, the integration of HS-SPME proves instrumental in enhancing chemical enrichments for OC analysis. The findings emphasize notable disparities in the distribution, morphology, and chemical composition of oil cells in Asari Radix et Rhizoma between roots and rhizomes, presenting a noteworthy phenomenon. The diverse chemical profiles observed across the four distinct types of oil cells suggest potential functional distinctions. Future investigations, including the transcriptomics analyses of different oil cell types, offer promise in unraveling the underlying mechanisms. This research serves as a valuable reference for the isolation and analysis of single plant cells.

## Materials and methods

### Preparation of plant material

The Asarum Root et Rhizoma used in this experiment was sourced from the dry roots and rhizomes of *Asarum heterotropoides* Fr. Schmidt var. *mandshuricum* (Maxim.) Kitag., collected in Liaoning, China. Voucher specimens (No. 20140807-(1)-SXYG) are deposited in the Herbarium of Pharmacognosy, School of Pharmaceutical Sciences, Peking University, China. After drying under shade, the samples were stored in dry, dark, sealed containers at room temperature. Fifty ARR herbs were randomly selected and softened in moist filter paper at 4 °C for 60–90 min. Dissecting blades were used to separate various parts of ARR, including fibrous roots, adventitious roots, rhizome piths, and rhizome cortexes. The rhizomes were dissected, and the pith and cortex were separated under a stereomicroscope (Leica, M165C, Germany). Adventitious roots and fibrous roots were cut to lengths of 1–2 cm (Fig. 1). All four parts were sampled and stored at 4 °C for later oil cell separation.

### Chemicals and reagents

The mixed standard n-alkanes (C_7_–C_30_, Lot: LC13543V) for calculating the retention index (RI) were obtained from Supelco company. Ten standards were utilized for chemical identification and methodology, including methyleugenol (Lot: PH3YH-MG), eucarvone (Lot: F1101-LHBN), l-borneol (Lot: EPH8L-QQ), safrole (Batch: 0452680-14), 3,4,5-trimethoxytoluene (Lot: 19923), 3,5-dimethoxytoluene (Lot: 10099004), 3,4-methylenedioxy propiophenone (Lot: M38410CCR0), elemicin (5-allyl-1,2,3-trimethoxybenzen, Lot: SY018605), kakuol (Lot: Y19J6H1), and 2,3,5-trimethoxytoluene (synthesized by our laboratory and identified by MS and NMR). The purity of all standards was higher than 97%. Chloral hydrate, phloroglucinol, hydrochloric acid, glycerol, and Sudan III were purchased from Tianjin Fuchen Chemical Reagent Factory (Tianjin, China), and Tissue-Tek OCT from Sakura Finetek (Nagano, Japan). Water was obtained from a Mili-Q water purification system (Millipore, Bedford, USA). All solvents were of chromatographic grade and were acquired from Fisher (Fair Lawn, NJ, USA).

### Micromanipulator and cell picking

The fibrous roots, adventitious roots, rhizome piths, and rhizome cortexes, each weighing 1 g, were collected and cut into small pieces approximately 2 mm in diameter. Using an electric homogenizer (IKA, T10, Germany), each tissue was homogenized 4–6 times (10 s per time, 1–2 min intervals) with 20 ml pure water added. The resulting tissue suspensions were filtered through 300-mesh and 80-mesh cell sieves to eliminate large tissue clumps and small cell debris. The filtered suspensions were then transferred to a big water droplet in 60 mm sterile Petri dishes, where numerous OCs and other residues were present. OCs, characterized by their glistening, spherical shape, were easily discernible under the microscope. A micromanipulator (Nikon, NT88 V3, Japan), comprising an inverted microscope and a micropipette on an electromechanical platform, was employed to directly hold and transfer target OCs from the suspension. For piping the contents of OCs, a micromanipulation needle combined with a micro pump was used (Fig. 2B2). Due to the thickened cell walls of OCs [44, 64], the needle hardly passed through the cell wall, and the contents were mostly in the semisolid state, making suction extraction challenging.

To overcome this challenge, a hand-made single-cell picking device was ingeniously employed for the successful transfer of OCs under 80 × magnification of the stereomicroscope. The cell-picking device was crafted by connecting a glass straw, rubber stopper, 5 ml syringe, cotton, yellow hose, and pipette tip with a filter. The device allowed for the collection of cells into straw via mouth pipetting [66]. The glass straw, with tip diameters of approximately 200 μm, was prepared by drawing 1 mm apart diameter glass tubes over an alcohol blast burner and breaking away the tip of the melted glass until an opening was formed (Fig. 2A). Ten OCs were collected at once and placed in a droplet of 200 μl 20% NaCl aqueous solution. OCs from the fibrous roots, adventitious roots, rhizome pith, and cortex of six ARR samples were sampled in parallel and labeled as XW1-6, XG1-6, SUI1-6, and PI1-6 respectively (Fig. 3), and stored at 4℃ for subsequent testing.

### Laser capture microdissection

LCM tissue sampling from the dried herbs followed previously established protocols [70, 71], but without the use of a nonfluorescent polyethylene terephthalate (PET) (Fig. 2C). ARR samples were directly sectioned to approximately 50 μm thickness using a cryotome (Leica, CM1860, Germany). The sections were placed on a steel frame without a PET membrane (Leica Microsystems, 76 × 26 mm, Germany), with a small portion of the section resting on a manually constructed support and a large portion left suspended. The Leica LMD 7000 system, operating under fluorescence mode with a dichromatic mirror, was employed to capture oil cells. The optimized microdissection conditions included a DPSS laser beam at 349 nm wavelength, a speed of 12, power of 50–60 μJ, and an aperture of 10 under a Leica LMD-BGR fluorescence filter system at × 6.3, × 10, or × 20/40 magnification. Captured cells fell into a cap of 500 μl microcentrifuge tube (Leica Microsystems) through gravity. Within the tube, 100 μl of a 20% NaCl aqueous solution has been added to hold the residue cells. Subsequently, the tube was centrifuged (Eppendorf, Centrifuge 5424R, Germany) at 10,000 rpm for 5 min. The cells, along with the solution, were then transferred using a pipette (Eppendorf, 1000 μl, Germany) into 10 ml gas phase vials. To ensure the collection of intact cells, the tube was washed two times with 100 μl solution, and this process was carefully examined under a stereo microscope. A total of 50 OCs were collected for each sample.

### Light microscopy and confocal laser scanning microscopy (CLSM)

For the histochemical study, sections of the rhizome, adventitious roots, and fibrous roots were obtained using a cryotome (Leica, CM1860, Germany). Viscous dyes and reagents were applied to these sections: Sudan III (Johansen, 1940) was used for lipids in OCs, while phloroglucinol was utilized to detect lignin, revealing the thickened cell wall of OCs and other cells. Isolated OCs were immersed in pure water to assess cellular integrity through both light microscopy (Olympus, BX53, Japan) and CLSM (Nikon, A1, Japan). The autofluorescence of OC walls, attributed to the presence of suberin and lignin [44], facilitated the visualization of their shape using CLSM. Observations were made using 20 × and 40 × lenses with an additional 10 × zoom. CLSM images were captured using the NIS-Elements AR software (Nikon) at a resolution of 1024 × 1024 pixels. Excitation was achieved with a 488.2 nm argon laser, and detection of fluorescence employed a Galvano Scanner, DU4 detector, and three filters (450/50, 525/50, 595/50 nm).

### HS–SPME–GC–MS condition

Chemical analyses were conducted using HS–SPME–GC–MS (Shimadzu, QP-2010 Ultra, Japan) combined system, featuring an AOC-5000 automatic sampler for solid-phase microextraction injection (Supelco, SPME fiber assembly 65 μm PDMS/DVB, USA) and static, lipid headspace (Hamilton, 2.5 ml headspace syringe, Germany). Chromatographic separations employed a VF-WAXms capillary column (Agilent, CP9205, 30 m × 0.25 mm, 0.25 μm coating thickness, Germany), with sampling from 10 ml headspace bottles (GL Science, Japan) equipped with a magnetic cap and silicone/PTFE septum.

The GC–MS method was optimized for improved chemical separation with the following parameters. The column temperature was programmed as follows: 0 min at 40 °C, 5 °C/min to 100 °C and holding for 10 min, 5 °C/min to 110 °C and holding for 5 min, 5 °C/min to 190 °C, 10 °C/min to 130 °C and holding for 6 min. High-purity helium served as the carrier gas at a column flow of 1.2 ml/min, with a split ratio of 1:1, and an injection temperature of 230 °C. The spectrometers were operated in the electron-impact (EI) mode, with a scan range was m/z 35–500, a scan rate of 0.30 s per scan, and an ionization energy of 70 eV. The ion source and interface temperature were set at 200 °C and 230 °C respectively. The temperature and time of extraction (PDMS/DVB) were set at 70 ℃ for 30 min extraction of violent compounds in the OCs, followed by desorption at 250 ℃ for 3 min, utilizing a 2.5 mL syringe of headspace and a sampling time of 0.5 min.

### Data analysis

The Shimadzu GC–MS solution workstation (Version 4.45) was used to analyze the MS data using standard substances, the NIST14 library, and relevant literature. The retention index (RI) for each compound was calculated using a mixed standard n-alkanes (C_7_–C_30_) [72, 73], and compared with literature values and the NIST Chemistry Web-Book. XCMS was utilized for aligning retention times and screening different ions among the samples. In this process, GC–MS data in an acceptable format (.CDF) was converted and uploaded to the XCMS-online system. The charge-to-mass ratio and retention time of each ion were exported to Excel. Then zero-intensity ions were filtered out to achieve specific ions among different samples. The resulting data without zero-intensity ions were subjected to statistical analysis using SMICA-P 14.0 software, including PCA, OPLS-DA, and PLS-DA. Ions with VIP values greater than one indicated components of significant differences, and ion intensity was used as a semi-quantitative indicator for relative content comparison among these OCs. Additionally, the area normalization method was also used to compare the relative contents of the volatile constituents in each sample.

### Supplementary Information

**Additional file 1.** Total ion chromatogram (TIC) of samples obtained by LCM from the adventitious roots of Asari Radix et Rhizoma.

## Data Availability

The datasets used during the current study are available from the corresponding author on reasonable request.
