# Supplementary material for: The isolation strategy and chemical analysis of oil cells from Asari Radix et Rhizoma
Source: Plant Methods. 2024 May 17;20:72. doi: 10.1186/s13007-024-01184-5 (PMC11100110; doi:10.1186/s13007-024-01184-5)

Additional file 1: Total ion chromatogram (TIC) of samples obtained by LCM from the adventitious roots of Asari Radix et Rhizoma. ARR. XGA: epidermis, XGB: cortex, XGC: phloem, XGE: xylem, XGO: oil cells in cortex

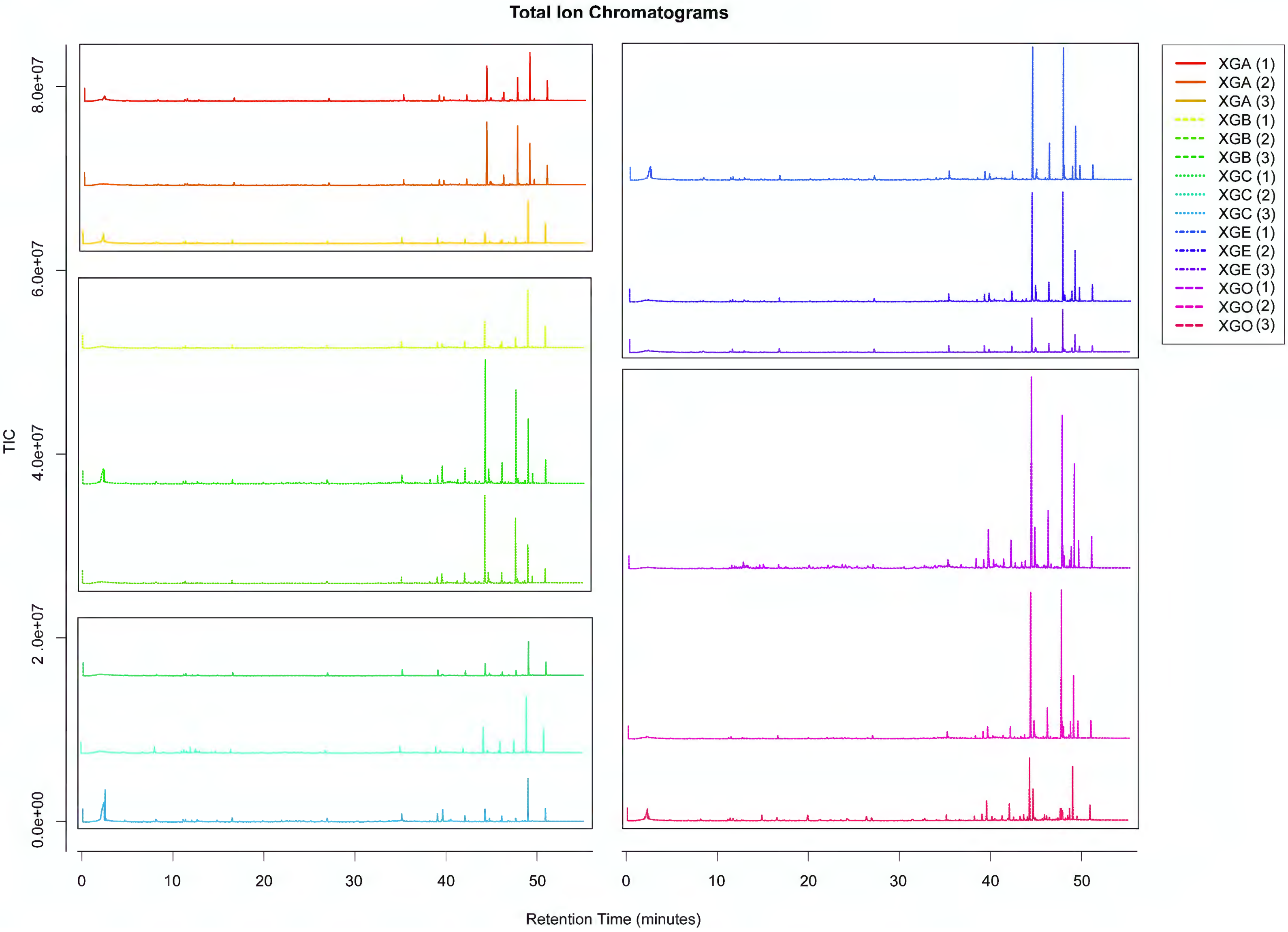

Supplement: Supplementary file 1 — Additional file 1. Total ion chromatogram (TIC) of samples obtained by LCM from the adventitious roots of Asari Radix et Rhizoma. [file 13007_2024_1184_MOESM1_ESM.pdf]
